# Diagnostic accuracy of point-of-care ultrasound for shock: a systematic review and meta-analysis

**DOI:** 10.1186/s13054-023-04495-6

**Published:** 2023-05-25

**Authors:** Takuo Yoshida, Takuya Yoshida, Hisashi Noma, Takeshi Nomura, Akihiro Suzuki, Takahiro Mihara

**Affiliations:** 1 Department of Health Data Science, Graduate School of Data Science, Yokohama City University, 22-2 Seto, Kanazawa, Yokohama, 236-0027 Japan; 2 Department of Emergency Medicine, Jikei University School of Medicine, Minato-ku, 105-8471 Japan; 3 Department of Data Science, The Institute of Statistical Mathematics, Tachikawa, 190-8562 Japan; 4 Department of Perioperative Medical Support, Tokushukai Medical Corporation, Chiyoda-ku, 102-0074 Japan; 5 Department of Anesthesiology and Critical Care Medicine, Jichi Medical University, Shimotsuke, 329-0498 Japan; 6 Department of Anesthesiology, Yokohama City University School of Medicine, Yokohama, 236-0004 Japan

**Keywords:** Circulatory failure, Shock, Point-of-care ultrasound, Diagnostic accuracy, Obstructive shock, Early diagnosis, Systematic review

## Abstract

**Background:**

Circulatory failure is classified into four types of shock (obstructive, cardiogenic, distributive, and hypovolemic) that must be distinguished as each requires a different treatment. Point-of-care ultrasound (POCUS) is widely used in clinical practice for acute conditions, and several diagnostic protocols using POCUS for shock have been developed. This study aimed to evaluate the diagnostic accuracy of POCUS in identifying the etiology of shock.

**Methods:**

We conducted a systematic literature search of MEDLINE, Cochrane Central Register of Controlled Trials, Embase, Web of Science, Clinicaltrial.gov, European Union Clinical Trials Register, WHO International Clinical Trials Registry Platform, and University Hospital Medical Information Network Clinical Trials Registry (UMIN-CTR) until June 15, 2022. We followed the Preferred Reporting Items for Systematic Reviews and Meta-Analyses guidelines and assessed study quality using the Quality Assessment of Diagnostic Accuracy Studies 2 tool. Meta-analysis was conducted to pool the diagnostic accuracy of POCUS for each type of shock. The study protocol was prospectively registered in UMIN-CTR (UMIN 000048025).

**Results:**

Of the 1553 studies identified, 36 studies were full-text reviewed, and 12 studies with 1132 patients were included in the meta-analysis. Pooled sensitivity and specificity were 0.82 [95% confidence interval (CI) 0.68–0.91] and 0.98 [95% CI 0.92–0.99] for obstructive shock, 0.78 [95% CI 0.56–0.91] and 0.96 [95% CI 0.92–0.98] for cardiogenic shock, 0.90 [95% CI 0.84–0.94] and 0.92 [95% CI 0.88–0.95] for hypovolemic shock, and 0.79 [95% CI 0.71–0.85] and 0.96 [95% CI 0.91–0.98] for distributive shock, respectively. The area under the receiver operating characteristic curve for each type of shock was approximately 0.95. The positive likelihood ratios for each type of shock were all greater than 10, especially 40 [95% CI 11–105] for obstructive shock. The negative likelihood ratio for each type of shock was approximately 0.2.

**Conclusions:**

The identification of the etiology for each type of shock using POCUS was characterized by high sensitivity and positive likelihood ratios, especially for obstructive shock.

**Supplementary Information:**

The online version contains supplementary material available at 10.1186/s13054-023-04495-6.

## Background

Circulatory failure is a syndrome that should be diagnosed and treated early [1–3]. It is classified into four types of shock: obstructive, cardiogenic, distributive, and hypovolemic, each of which must be treated differently [1]. Therefore, when encountering circulatory failure, it is important to differentiate the type of shock the patient is experiencing. Clinically, shock is differentiated using all available information, including medical history, blood tests, and various imaging studies. Of these, performing an ultrasound, in particular, has the potential to directly delineate and identify the etiology of shock [4–9]. Furthermore, sometimes multiple shocks can overlap, making diagnosis difficult [1]. Therefore, ultrasonography, which allows direct and rapid observation of the pathophysiology with images [7], may be crucial for the management of shock.

The rapid bedside diagnosis of the etiology of an acute condition using ultrasonography is called point-of-care ultrasound (POCUS) [7] and has attracted considerable attention in recent years [10–13]. Several diagnostic protocols have been proposed for POCUS for shock [14–19]. In the standard cardiac view (parasternal long- and short-axis, apical four-chamber, and subcostal four-chamber), qualitative assessment of left and right ventricle size and contractile function, and physiologic assessment of pericardial fluid and tamponade were common to all protocols [14–19]. Their common feature was early bedside goal-directed diagnosis by ultrasound. A systematic review of the diagnostic accuracy of POCUS for shock was reported in 2019 [20]. However, the study was limited to a meta-analysis of only four small observational emergency room studies. Moreover, despite the rapid increase in literature dealing with POCUS in emergency and intensive care settings [10], to the best of our knowledge, no systematic review summarizing the diagnostic accuracy for each type of shock has been reported. Furthermore, although the POCUS protocols for shock have described the findings and rough differentiation steps for shock [20, 21], the specific order and site of ultrasound examination have not been clearly established. These factors should be considered when considering the differences in the diagnostic accuracy for each type of shock.

Therefore, to address these uncertainties, we conducted a systematic review and meta-analysis to assess the accuracy of POCUS for the diagnosis of shock among adult patients with circulatory failure.

## Methods

In this study, we adhered to the Cochrane Handbook for Diagnostic Test accuracy [22] and reported according to the Preferred Reporting Items for Systematic Review and Meta-analysis of Diagnostic Test Accuracy Studies (PRISMA-DTA) guidelines [23, 24]. The study protocol is registered at University Hospital Medical Information Network Clinical Trials Registry (UMIN-CTR) (UMIN 000048025). In this study, we defined POCUS with echocardiography, which was immediately performed in shock patients for diagnosis of the cause of circulatory failure, as the index test. There were no restrictions regarding where POCUS was conducted. As there is no specific diagnostic method for differentiating the cause of shock [1], we defined the clinical diagnosis based on the medical information available within each study as the reference standard. The location and timing of clinical diagnosis needed to be different from any of those in which POCUS was performed. The target condition of interest was circulatory failure with no identified etiology, and the definition of circulatory failure was based on the definition in each study.

### Data sources and searches

A computerized search of the electronic databases of MEDLINE, Cochrane Central Register of Controlled Trials (CENTRAL), Embase, Web of Science, Clinicaltrials.gov, European Union Clinical Trials Register (EU-CTR), WHO International Clinical Trials Registry Platform (ICTRP), and UMIN-CTR was performed from inception of the databases to June 15, 2022. Moreover, we manually searched the reference lists of the relevant articles. Searches involved a combination of free-text words and MeSH (Medical Subject Headings) terms using permutations of the search terms “intensive care,” “critical illness,” “emergencies,” “point of care,” “focus,” “ultrasound,” “echocardiography,” “shock,” “hypotension,” and “circulatory failure” (Additional file 1: Table S1). Methodological search filters were avoided. The results from all languages were included. Furthermore, we also included abstracts presented at national and international conferences if they were published in journal supplements after the conference.

### Study selection

We included prospective and retrospective observational studies, and secondary analyses of randomized controlled trial data reporting the diagnostic accuracy of POCUS for the diagnosis of etiology in adult patients (≥ 18 years old) with undifferentiated shock. There were no limitations on the language or publication date for this review. Moreover, we excluded diagnostic case–control studies (two-gate studies) and case studies that lacked DTA data, namely true-positive, false-positive, true-negative, and false-negative values. Two reviewers independently screened the titles and abstracts of all eligible studies to identify candidates for full-text review. The articles selected for full-text review were then independently reviewed to identify those appropriate for inclusion. Disagreements between the reviewers were resolved through discussion or by a third reviewer. If multiple published studies were identified based on the same database, the most recent and complete studies were included in the analysis.

### Data extraction and quality assessment

Two authors independently extracted data and assessed study quality and applicability using the QUADAS-2 (Quality Assessment of Diagnostic Accuracy Studies 2) tool [25], which includes four risk-of-bias domains (patient selection, index test, reference test, and flow and timing) and three domains of applicability (patient selection, index test, and reference test). Disagreements between the reviewers were resolved through discussion and consensus. The following data were extracted using a pre-defined data extraction form: study characteristics (author, year of publication, country, design, sample size, clinical settings, conflict of interest, and funding source), patient characteristics (inclusion/exclusion criteria and patient clinical and demographic characteristics), index test (timing of diagnosis, protocol of ultrasound, and the person who conducted the test), reference standard (timing of diagnosis, information referred for diagnosis, and the person who conducted the diagnosis), and diagnostic accuracy parameters (the rates of true-positive, true-negative, false-positive, and false-negative results for each pair of index tests and target conditions). If the original manuscript did not contain sufficient relevant data on diagnostic accuracy, we contacted the authors of the paper to request additional data or to incorporate any available data from previous systematic reviews into the analysis. All data were extracted independently and in duplicate, and any differences between the reviewers were resolved by consensus.

### Data synthesis and analysis

For each shock category, we calculated the sensitivity and specificity of the index individual studies with corresponding 95% confidence intervals (CIs) and plotted them on forest plots to assess heterogeneity. Synthesis analyses were performed using Reitsma’s bivariate random-effects model [26] for study-specific sensitivities, specificities, positive likelihood ratios, and negative likelihood ratios, considering possible heterogeneities across the studies. We evaluated summary estimates, and calculated their inconsistencies (*I*^2^), which described the percentage of total variation across studies due to heterogeneity rather than chance. To visually evaluate the variability in the diagnostic accuracy of POCUS for each shock, we created summary receiver operating characteristic (SROC) curves for each shock [27] based on the estimates of the bivariate random effects model and presented the areas under the curves (AUCs) of the SROC curves as summary measures of the predictive accuracy measures [28]. For statistical inferences, we used the standard restricted maximum likelihood estimation for the Reitsma’s model, and the bootstrap method to calculate the 95% CIs of the AUCs of the SROC curves. In addition, we performed subgroup analyses for each shock based on the following variables that were assumed to influence diagnostic accuracy estimates: suspected disease before POCUS, presence of ultrasound other than echocardiography, settings in which ultrasound was performed, and a training program for point-of-care ultrasound. We also performed sensitivity analysis after excluding studies with a high risk of bias. Publication or reporting bias was not assessed because there is no accepted method that can be used for its evaluation in a meta-analysis of diagnostic test accuracy studies [29–31]. All statistical analyses were performed using R version 4.1.2 (The R Foundation for Statistical Computing, Vienna, Austria).

## Results

**Fig. 1:**
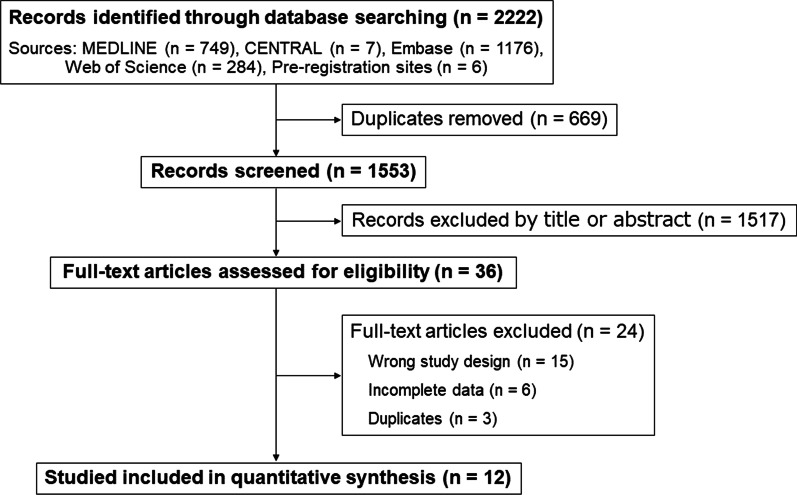
Study flow diagram. *CENTRAL* Cochrane Central Register of Controlled Trials

We screened the titles and abstracts of 1553 studies and reviewed 36 full-text articles after excluding studies that were non-diagnostic, clearly unrelated to POCUS, or did not meet the inclusion criteria for this study (Fig. 1). In the full-text review, the number of true positives, true negatives, false positives, or false negatives for 15 studies was unavailable due to the wrong study design. For six studies, the contact information was unavailable, or the authors were contacted, but complete data were unavailable. The list of excluded studies is shown in Additional file 1: Table S2. Finally, twelve studies with 1132 patients with shock [32–43] were identified as eligible for meta-analysis, as well as an additional six that met the inclusion criteria but provided insufficient data. In one study [43], the numbers of true-positive, true-negative, false-positive, and false-negative results reported in a previous systematic review were used because of insufficient data for diagnostic accuracy.

The baseline characteristics of the eligible studies are presented in Table 1 and Additional file 1: Table S3; eleven of these were prospective cohort studies. Although the detailed definition of shock differed in each study, the occurrence of hypotension was common to all studies. With regard to the index test, all but one study [37] used POCUS, which consists of multiple-organ ultrasound, including echocardiography. In almost all studies, the reference standard was defined as a clinical diagnosis based on medical records. Two observational studies had prior suspected disease in a group of patients with suspected pulmonary embolism [35, 37].

**Table 1:** Study characteristics

Study	Design, no. of patients (location)	Clinical setting	Definition of circulatory failure	US protocol	US Physician	Reference standard
Bagheri-Hariri et al. [40]	Prospective Cohort, one center, 25 patients (Iran)	Emergency department	SBP < 90 mmHg or shock index^a^ > 1.0 with clinical hypoperfusion symptoms	Multi-organ POCUS (observed in order: heart/IVC, jugular veins, thoracic and abdominal cavities, lungs/deep veins, aorta)^b^	Emergency physicians with credentials for the emergency department ultrasound	Clinical diagnosis using all medical information
Ghane et al. [33]	Prospective Cohort, one center, 77 patients (Iran)	Emergency department	SBP < 100 mmHg or shock index^a^ > 1.0	Multi-organ POCUS (observed in order: heart/IVC, jugular veins, thoracic and abdominal cavities, lungs/deep veins, aorta) ^b^	An emergency physician with five years of experience with more than 200 ultrasonographic exams per year	Clinical diagnosis after admission to the medical units (internal medicine, cardiology, or surgery) by board-certified specialists
Shokoohi et al. [43]	Prospective Cohort, one center, 118 patients (USA)	Emergency department	SBP < 90 mmHg after an initial fluid resuscitation (> 1L of normal saline)	Multi-organ POCUS (no order specified: heart, IVC, thoracic and abdominal cavities, and lung)	An ultrasound-trained attending physician (including ultrasound fellows) with extensive experience in emergency and critical care ultrasound	Clinical diagnosis by chart review by two board-certified intensivists, blinded to the results of POCUS
Agmy et al. [41]	Unknown, one center, 63 patients (Egypt)	Intensive care unit	Circulatory shock patients (definition was unknown)	Multi-organ POCUS (observed in order: heart and lung)^c^	Unclear	Clinical diagnosis using all medical information
Nazerian et al. [35]	Prospective Cohort, two center, 105 patients (Italy)	Emergency department	SBP < 90 mmHg or a drop of SBP > 40 mmHg for more than 15 min, with signs of end-organ hypoperfusion (cold extremities, UO < 30 mL/h, altered mental status, profound asthenia with fatigue and malaise, or respiratory distress), with suspected PE	Multi-organ POCUS (no order specified: heart and deep veins)	Sonographers with more than 2 years’ experience in cardiac and venous US on critically ill patients	Clinical diagnosis by an expert in PE who independently reviewed all the available clinical and imaging data including multidetector computed tomography pulmonary angiography
Elbaih et al. [38]	Prospective Cohort, one center, 100 patients (Egypt)	Emergency department	Unstable polytrauma patients (definition of unstable was unknown)	Multi-organ POCUS (observed in order: heart/IVC, jugular veins, thoracic and abdominal cavities, lungs/deep veins, aorta)^b^	Unclear	Clinical diagnosis using all medical information
Tesfaye et al. [42]	Prospective Cohort, one center, 93 patients (Ethiopia)	Emergency department	Hypotension (definition of hypotension was unknown)	Multi-organ POCUS (observed in order: heart/IVC, jugular veins, thoracic and abdominal cavities, lungs/deep veins, aorta)^b^	Unclear	Clinical diagnosis after full evaluation
Daley et al. [37]	Prospective Cohort, six centers, 136 patients (USA)	Emergency department	Tachycardia and/or hypotension with suspected PE (definition of tachycardia and hypotension was unknown)	Heart including the measurement of TAPSE^d^	Emergency physicians or study investigators (including medical students) trained in FOCUS	Computed tomography angiography
Rahulkumar et al. [36]	Prospective Cohort, one center, 97 patients (India)	Emergency department	SBP < 90 mmHg and shock index^a^ > 1.0	Multi-organ POCUS (observed in order: heart/IVC, jugular veins, thoracic and abdominal cavities, lungs/deep veins, aorta) ^b^	An emergency physician expert in emergency medicine ultrasound	Clinical diagnosis using all medical information by the consultants of medicine or surgery department
Javali et al. [39]	Prospective Cohort, one center, 100 patients (India)	Emergency department	SBP < 90 mmHg and shock index ^a^ > 1 with the presence of at least one of the following signs or symptoms of hypoperfusion unresponsiveness, altered mental status, syncope, respiratory distress, generalized fatigue, severe chest pain or abdominal pain	Multi-organ POCUS (no order specified: heart, lung, free fluid in the peritoneal cavity, aorta, IVC, and femoral vein)	A trained emergency physician (unclear regarding ultrasound experience)	Clinical diagnosis after admission to the medical units (internal medicine, cardiology, or surgery) by board-certified specialists, blind to the diagnoses in the emergency department
Keefer et al. [32]	Prospective Cohort, six centers, 135 patients (North America and South Africa)	Emergency department	Sustained SBP < 100 mmHg or shock index^a^ > 1.0	Multi-organ POCUS (observed in order: heart/IVC, jugular veins, thoracic and abdominal cavities, lungs/deep veins, aorta) ^b^	POCUS-trained emergency physicians	Clinical diagnosis by chart review by two clinicians, blinded to the initial sonographer, and point-of-care ultrasonography findings and diagnosis
Zieleskiewicz et al. [34]	Prospective Cohort, one center, 83 patients (France)	General ward	MAP < 65 mmHg or HR < 40 bpm or HR > 120 bpm or UO < 50 ml/4 h	Multi-organ POCUS (no order specified: heart, IVC, lung, thoracic cavity, and the deep veins if required)	ICU physicians trained in ultrasound	Clinical diagnosis by chart review including physical examinations and blood and imaging tests by two physicians blinded of the initial diagnoses made at the bedside

SBP, systolic blood pressure; MAP, mean arterial pressure; HR, heart rate; bpm, beat per minutes; UO, urine output; PE, pulmonary embolism; US, ultrasound; TAPSE, tricuspid annular plane systolic excursion; and IVC, inferior vena cava

^a^Shock index is defined as the heart rate divided by systolic blood pressure

^b^Described as RUSH (rapid ultrasound for shock and hypotension) exam in this study

^c^Described FALLS (fluid administration limited by lung sonography) protocol in this study

^d^Described FOCUS (focused cardiac ultrasound) in this study

The results of the QUADAS-2 assessment are shown in Table 2. Three studies were judged to have a high risk of bias in the patient selection domain because the inclusion processes were not conducted consecutively or randomly [33, 38, 39]. In one study, POCUS was performed after the implementation of the reference standard and was considered to have a high risk of bias in the domain of the index test because it may have influenced the interpretation of the results of the index test [37]. In the domain of flow and timing in three studies [32, 33, 35], the risk of bias was considered high because of the presence of cases that were excluded from the analysis for unknown reasons. For two studies, for which only conference abstracts were available [41, 42], the detailed definitions of the inclusion criteria were unclear, as were the details of the number of patients involved in the process, from patient selection to analysis. Additionally, the risk of bias with respect to applicability was unknown because the patient populations included were unclear.

**Table 2:** QUADAS-2 results

Study	Risk of bias				Applicability concerns		
	Patient selection	Index test	Reference standard	Flow and timing	Patient selection	Index test	Reference standard
Bagheri-Hariri et al. [40]	Low	Low	Low	**High**	Low	Low	Low
Ghane et al. [33]	**High**	Low	Low	**High**	Low	Low	Low
Shokoohi et al. [43]	**High**	Low	Low	Low	Low	Low	Low
Agmy et al. [41]	*Unclear*	Low	Low	*Unclear*	*Unclear*	Low	Low
Nazerian et al. [35]	Low	Low	Low	**High**	Low	Low	Low
Elbaih et al. [38]	**High**	Low	Low	Low	Low	Low	Low
Tesfaye et al. [42]	*Unclear*	Low	Low	*Unclear*	*Unclear*	Low	Low
Daley et al. [37]	Low	**High**	Low	Low	Low	Low	Low
Rahulkumar et al. [36]	Low	Low	Low	Low	Low	Low	Low
Javali et al. [39]	**High**	Low	Low	Low	Low	Low	Low
Keefer et al. [32]	Low	Low	Low	**High**	Low	Low	Low
Zieleskiewicz et al. [34]	Low	Low	Low	Low	Low	Low	Low

**Fig. 2:**
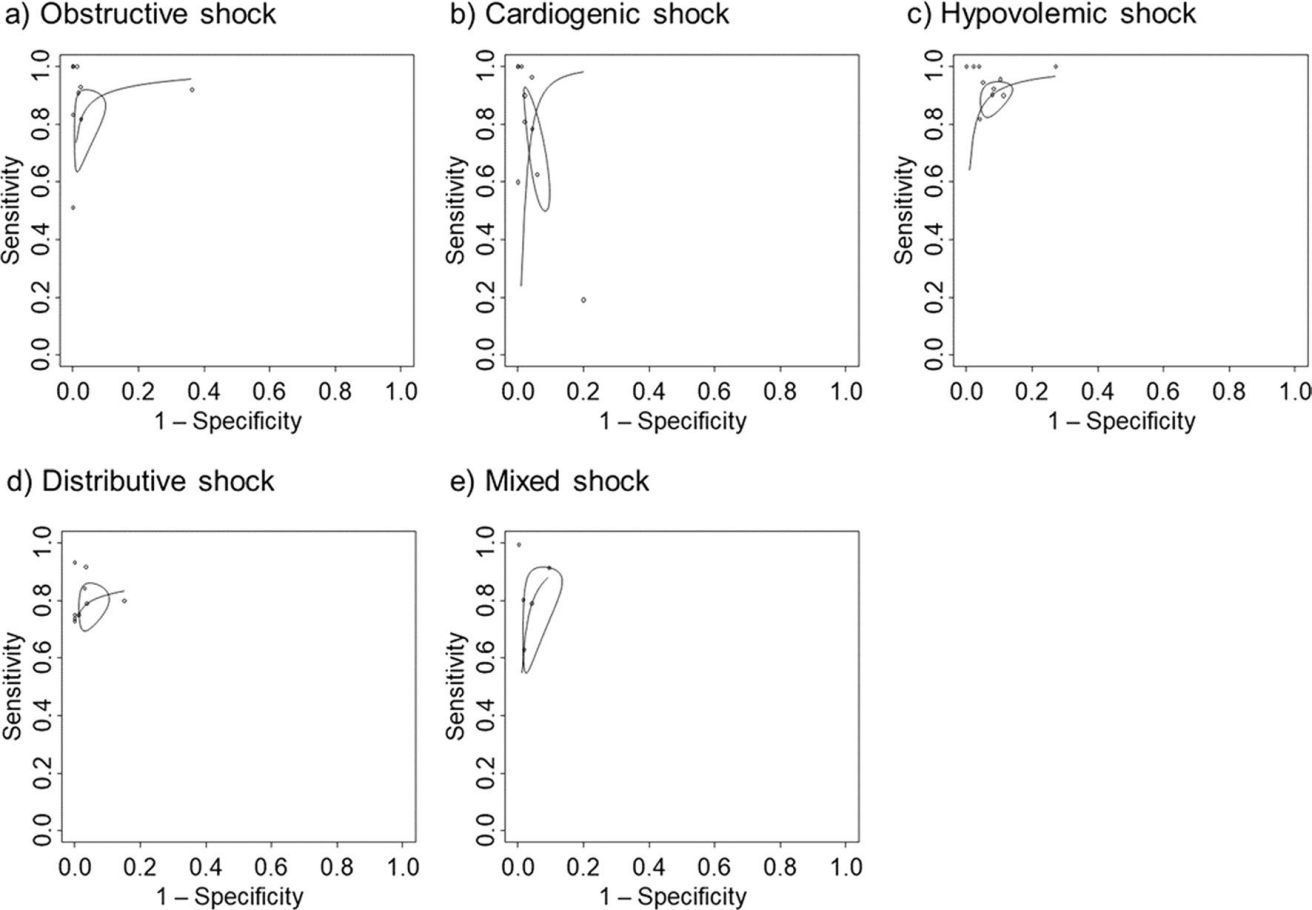
Summary receiver operating characteristic curves. The summary receiver operating characteristic plots of the bivariate meta-analysis for the identification of the cause of shock by point-of-care ultrasound. The ellipse around the point estimates represents a 95% CI. The ROC curves are restricted to the range of specificities for each study

The SROC curves relevant to each shock are shown in Fig. 2, and summary estimates of sensitivity, specificity, AUC of the SROC curve, positive likelihood ratio, and negative likelihood ratio are shown in Table 3. In Fig. 2, the solid curves are the SROC curves integrated into a bivariate random-effects model for diagnosing the cause of each shock using POCUS. The dots represent point estimates of sensitivity and 1-specificity for each included study, and the ellipses represent 95% CIs for sensitivity and 1-specificity. The AUCs of the SROC curves for each shock were approximately 0.95, except for distributive shock. Point estimates of sensitivity for each shock ranged from approximately 0.8 to 0.9 with wide CIs, and point estimates of specificity exceeded 0.9 for all shocks with narrow CIs. For all shocks, the positive likelihood ratios were generally above 20, and the negative likelihood ratios were approximately 0.2. Among these shocks, the specificity and positive likelihood ratio for obstructive shock were particularly high. The *I*^2^ value, a measure of heterogeneity, was 15.1% for obstructive shock and 0% for all others. Forest plots for each shock are shown in Additional file 1: Figures S1 to S5.

**Table 3:** Sensitivities, specificities, AUROCs, and likelihood ratios by shock subtype

Shock type	No. of patients (study)	Sensitivity	Specificity	Area under the ROC curve	Positive likelihood ratio	Negative likelihood ratio
Obstructive	810 (9)	0.82 (0.68–0.91)	0.98 (0.92–0.99)	0.95 (0.78–0.97)	40 (11–105)	0.20 (0.10–0.33)
Cardiogenic	828 (9)	0.78 (0.56–0.91)	0.96 (0.92–0.98)	0.96 (0.86–0.97)	19 (7.1–40)	0.24 (0.09–0.47)
Hypovolemic	688 (9)	0.90 (0.84–0.94)	0.92 (0.88–0.95)	0.96 (0.87–0.96)	12 (7.3–18)	0.11 (0.07–0.17)
Distributive	594 (8)	0.79 (0.71–0.85)	0.96 (0.91–0.98)	0.86 (0.75–0.96)	23 (9.3–49)	0.22 (0.16–0.30)
Mixed	291 (4)	0.80 (0.61–0.91)	0.96 (0.89–0.99)	0.95 (0.76–0.97)	20 (7.9–49)	0.21 (0.10–0.40)

As a subgroup analysis, we performed a meta-analysis of studies that were only performed in the emergency room [32, 34–40, 42, 43], those that had no suspected disease before POCUS [32–34, 36, 38–43], clearly stated the existence of a POCUS training program [32–34, 37], or that had a POCUS protocol for multi-organ ultrasound [32–36, 38–43]. The results are shown in Additional file 1: Tables S4 to S7. The results of the meta-analysis of the studies performed in the emergency room were similar to those of the main analyses. The results of the meta-analysis in the absence of suspected disease before POCUS and multi-organ ultrasonography showed a noticeably higher specificity and positive likelihood ratio for obstructive shock than the results of the main analysis. Results from studies with POCUS training programs were notable for their high sensitivity, specificity, and likelihood ratio for distributional abnormal shocks; however, the number of studies included in this meta-analysis was small. As a sensitivity analysis, a meta-analysis was performed of the remaining ten studies after excluding two studies for which only conference abstracts were available [41, 42]. These results were similar to those of the main analysis (Additional file 1: Table S8).

## Discussion

This systematic review included 12 studies with 1132 patients with shock and evaluated the diagnostic accuracy of POCUS in diagnosing the etiology. Compared to the previous systematic review that addressed the same topic [20], this review, which was updated with an expanded population, was able to incorporate more studies and thus present a narrower confidence interval for each diagnostic accuracy. In addition, the various subgroup analyses confirmed the characteristics of the diagnostic accuracy of POCUS for shock patients. The meta-analysis we conducted showed pooled sensitivity for each type of shock ranged from 0.77 (distributive) to 0.93 (hypovolemic) and specificity ranged from 0.92 (hypovolemic) to 0.97 (obstructive), and the area under the ROC for each type was approximately 0.95. Positive likelihood ratios exceeded 10 for all types of shocks, especially obstructive, and negative likelihood ratios were about 0.2 for each.

Several systematic reviews have been conducted on the diagnostic accuracy of POCUS in emergency conditions. A systematic review of POCUS for respiratory failure reported a sensitivity of 0.92 (95% CI 0.85–0.96) and a specificity of 0.98 (95% CI 0.94–0.99) [44]. Another systematic review for the detection of signs of significant injury in thoracoabdominal trauma (thoracoabdominal fluid retention, large vessel injury, pneumothorax, etc.), including 34 studies and 8635 patients published by Cochrane in 2018, found a sensitivity of 0.74 (95% Cl: 0.65–0.81) and a specificity of 0.96 (95% Cl: 0.94–0.98) [45]. Furthermore, with regard to POCUS in shock differentiation, the previous systematic review showed that the diagnostic accuracies for each type of shock ranged from 0.64–0.93 for sensitivity, 0.80–0.98 for specificity, 8–40 for positive likelihood ratio, and 0.13–0.32 for negative likelihood ratio [20]. Compared to these studies, the characteristics of diagnostic accuracy for each type of shock in our systematic review were similar in terms of high specificity. Therefore, when POCUS detects a finding that could be the cause of shock, clinicians should also recognize it as a probable cause.

Clinically, the difficulty in identifying the cause of shock using POCUS may vary depending on the etiology. In a previous systematic review, a comparison between each type of shock showed high specificity and positive likelihood ratios, especially in obstructive shock (specificity 0.98 (95% CI 0.96–0.99) and positive likelihood ratio 40.54 (95% CI 12.06–136.28)) [20]. Another narrative review examining the diagnostic accuracies for acute diseases also showed particularly high specificity for pericardial effusion, right heart failure, and pneumothorax [1, 6, 46]. In our study, the specificity and positive likelihood ratios were the highest for obstructive shock. In addition, this trend was similar in the subgroup analyses. Clinically, obstructive shock often shows disease-specific findings on POCUS as the cause of shock. However, the echocardiographic findings of distributive and hypovolemic shock are identical, and even if cardiogenic shock is suspected due to apparent cardiac dysfunction, it is not known whether the cardiac dysfunction is new or contributes to shock. Therefore, the differential diagnosis of shock using POCUS is particularly useful for confirming obstructive shock. Furthermore, our subgroup meta-analysis, limited to studies using POCUS protocols for multiple organs, not only the heart, showed high diagnostic accuracy for obstructive shock, especially in terms of specificity and negative likelihood ratio. This may be because ultrasound findings other than echocardiography can rule out the causes of obstructive shock, such as lung sliding to rule out pneumothorax. It should be emphasized that POCUS should be performed on multiple organs in shock patients.

Among the diagnostic protocols for ultrasound in diagnosing the cause of shock, the Rapid Ultrasound for Shock and Hypotension (RUSH) examination is well known and has been used in many of the studies included in our meta-analysis [14]. The RUSH examination comprehensively described the ultrasound findings in multiple organs that were observed in each type of shock. However, to implement POCUS in actual clinical practice, it is necessary to provide more specific instructions on the method of performing it, such as the type of shocks to differentiate first and the view to start with in echocardiography. In our study, we found that the specificity and positive likelihood ratio of POCUS in identifying the cause of shock were high for all types of shock, particularly obstructive shock. Clinically, obstructive shock is a group of diseases that can be treated by eliminating confirmed abnormal findings. Therefore, when using POCUS to identify the cause of shock, it is reasonable to first confirm the diagnosis of obstructive shock, which may improve patient outcomes.

Although the basic level of POCUS in echocardiography requires the acquisition of parasternal long- and short-axis, apical four-chamber, subcostal four-chamber, and inferior vena cava (IVC) views [18, 19, 47, 48], the view with which it is initiated depends on the clinician’s discretion. Typical diseases that cause obstructive shock include tension pneumothorax, severe pulmonary embolism, and cardiac tamponade. Common echocardiographic findings in these diseases are dilation of the IVC and decreased respiratory variability in the IVC [14, 21, 49–53], which can be easily visualized from the subcostal four-chamber and IVC views. In addition, this view allows for quick assessment of cardiac tamponade by confirming the presence of pericardial fluid while viewing the IVC. Therefore, when differentiating shock using POCUS, it is appropriate to start with obstructive shock, given its diagnostic accuracy, and with the subcostal four-chamber view. This should be considered in future POCUS diagnostic protocols.

Our study included the largest number of studies on the diagnostic accuracy of POCUS for identifying the cause of shock. However, it has several limitations. First, of the 12 studies included, 11 used clinical diagnosis as the reference standard. Out of these studies, only four had blinding to the POCUS results during clinical diagnosis. Although there were no qualitative differences in diagnostic accuracy depending on whether the POCUS results were blinded, this may have led to an overestimation of the diagnostic accuracy of POCUS for shock patients. Second, there was no uniform definition of the reference standard among the included studies in terms of details beyond clinical diagnosis. However, there is no consistent diagnostic method for a definitive diagnosis of the cause of shock. In clinical practice, a definitive diagnosis is made by considering all medical information available on site. In general, the definition comprehensively defined as a clinical diagnosis in each study was considered to be in line with clinical practice. Third, in the index test, the details of POCUS (skill level of the performer and ultrasound protocol) were not consistently defined across all of the studies. However, the results of the meta-analysis of subgroup analyses were similar to those of the main analysis. Fourth, the studies included in our meta-analysis were conducted almost exclusively in emergency rooms. In other clinical settings, diagnostic accuracy may be altered by varying disease severity. For example, diagnostic accuracy in the intensive care unit, where more severely ill patients may be present, may differ from that of our study. However, further studies are required to address this issue. Finally, our study only examined the diagnostic accuracy, and it is unclear whether the use of POCUS truly improves patient outcomes. It is also unclear whether a better protocol would improve diagnostic accuracy or change actual practice. To resolve these uncertainties, further studies are needed to develop more clinically useful diagnostic protocols based on diagnostic accuracy and to examine the impact of protocol implementation on patient outcomes.

## Conclusions

In this study, the identification of the etiology of shock by POCUS was characterized by high sensitivity and a positive likelihood ratio, especially for obstructive shock. Hence, these findings should be considered in future diagnostic protocols for shock using POCUS. However, since this study only examined the diagnostic aspect, further interventional studies are necessary to assess the true impact of POCUS on shock patients.

## Supplementary Information

**Additional file 1: Table S1.** The full search strategy. **Table S2.** The list of excluded studies. **Table S3.** Additional study characteristics. **Table S4.** Diagnostic accuracies for studies conducted in the emergency department. **Table S5.** Diagnostic accuracies for studies with no prior suspected disease. **Table S6.** Diagnostic accuracies for studies in which the existence of a point-of-care ultrasound training program was explicitly mentioned. **Table S7.** Diagnostic accuracies for studies in which ultrasound other than transthoracic echocardiography was used in combination. **Table S8.** Diagnostic accuracies for studies without high risk of bias. **Figure S1:** Obstructive shock. CI, confidence interval. **Figure S2:** Cardiogenic shock. CI, confidence interval. **Figure S3:** Hypovolemic shock. CI, confidence interval. **Figure S4:** Distributive shock. CI, confidence interval. **Figure S5:** Mixed shock. CI, confidence interval.

## Data Availability

Data supporting the findings of this study are available from the corresponding author, Takuo Y, upon reasonable request.

## Abbreviations

POCUS: Point-of-care ultrasound
CENTRAL: Cochrane Central Register of Controlled Trials
EU-CTR: European Union Clinical Trials Register
ICTRP: International Clinical Trials Registry Platform
UMIN-CTR: University Hospital Medical Information Network Clinical Trials Registry
MeSH: Medical Subject Headings
QUADAS-2: Quality Assessment of Diagnostic Accuracy Studies 2
SROC: Summary receiver operating characteristic
AUCs: Area under the curve
CI: Confidence interval
IVC: Inferior vena cava
