# Supplementary material for: Diagnostic accuracy of point-of-care ultrasound for shock: a systematic review and meta-analysis
Source: Crit Care. 2023 May 25;27:200. doi: 10.1186/s13054-023-04495-6 (PMC10214599; doi:10.1186/s13054-023-04495-6)

**Table S1:** **Full Search Strategy**

| Database | Search term |
| --- | --- |
| MEDLINE | |
| 1 | "intensive care"[Title/Abstract] |
| 2 | "critical ill*"[Title/Abstract] |
| 3 | "critical care"[Title/Abstract] |
| 4 | "critical illness"[MeSH Terms] |
| 5 | "critical care"[MeSH Terms] |
| 6 | "intensive care units"[MeSH Terms] |
| 7 | "Critical Care Outcomes"[MeSH Terms] |
| 8 | "Critical Care Nursing"[MeSH Terms] |
| 9 | 1 or 2 or 3 or 4 or 5 or 6 or 7 or 8 |
| 10 | "Acute"[Title/Abstract] |
| 11 | "emergen*"[Title/Abstract] |
| 12 | "rapid*"[Title/Abstract] |
| 13 | "acute disease"[MeSH Terms] |
| 14 | "emergency service, hospital"[MeSH Terms] |
| 15 | "evidence based emergency medicine"[MeSH Terms] |
| 16 | "emergency medicine"[MeSH Terms] |
| 17 | "emergencies"[MeSH Terms] |
| 18 | "emergency nursing"[MeSH Terms] |
| 19 | "emergency treatment"[MeSH Terms] |
| 20 | "emergency medical services"[MeSH Terms] |
| 21 | "hospital rapid response team"[MeSH Terms] |
| 22 | 10 or 11 or 12 or 13 or 14 or 15 or 16 or 17 or 18 or 19 or 20 or 21 |
| 23 | 9 or 22 |
| 24 | "POCUS"[Title/Abstract] |
| 25 | "point-of-care"[Title/Abstract] |
| 26 | "point of care"[Title/Abstract] |
| 27 | "focus*"[Title/Abstract] |
| 28 | "FCU"[Title/Abstract] |
| 29 | "point of care testing"[MeSH Terms] |
| 30 | "point of care systems"[MeSH Terms] |
| 31 | "Protocol"[Title/Abstract] |
| 32 | 24 or 25 or 26 or 27 or 28 or 29 or 30 or 31 |
| 33 | "ultraso*"[Title/Abstract] |
| 34 | "echocardiogra*"[Title/Abstract] |
| 35 | "ultrasonography"[MeSH Terms] |
| 36 | "ultrasonics"[MeSH Terms] |
| 37 | 33 or 34 or 35 or 36 |
| 38 | "shock"[MeSH Terms] |
| 39 | "hypotension"[MeSH Terms] |
| 40 | "shock"[Title/Abstract] |
| 41 | "hypotension"[Title/Abstract] |
| 42 | "circulatory failure"[Title/Abstract] |
| 43 | "circulatory dysfunction"[Title/Abstract] |
| 44 | "circulatory disorder*"[Title/Abstract] |
| 45 | 38 or 39 or 40 or 41 or 42 or 43 or 44 |
| 46 | 23 and 32 and 37 and 45 |
|  |  |
| CENTRAL | |
| 1 | ("intensive care"):ti,ab,kw |
| 2 | ("critical ill"):ti,ab,kw (Word variations have been searched) |
| 3 | ("critical care"):ti,ab,kw |
| 4 | MeSH descriptor: [Critical Illness] this term only |
| 5 | MeSH descriptor: [Critical Care] this term only |
| 6 | MeSH descriptor: [Intensive Care Units] this term only |
| 7 | MeSH descriptor: [Critical Care Outcomes] this term only |
| 8 | MeSH descriptor: [Critical Care Nursing] this term only |
| 9 | #1 or #2 or #3 or #4 or #5 or #6 or #7 or #8 |
| 10 | (acute):ti,ab,kw |
| 11 | (emergen):ti,ab,kw (Word variations have been searched) |
| 12 | (rapid):ti,ab,kw (Word variations have been searched) |
| 13 | MeSH descriptor: [Acute Disease] this term only |
| 14 | MeSH descriptor: [Emergency Service, Hospital] this term only |
| 15 | MeSH descriptor: [Evidence-Based Emergency Medicine] this term only |
| 16 | MeSH descriptor: [Emergency Medicine] this term only |
| 17 | MeSH descriptor: [Emergencies] this term only |
| 18 | MeSH descriptor: [Emergency Nursing] this term only |
| 19 | MeSH descriptor: [Emergency Treatment] this term only |
| 20 | MeSH descriptor: [Emergency Medical Services] this term only |
| 21 | MeSH descriptor: [Hospital Rapid Response Team] this term only |
| 22 | #10 or #11 or #12 or #13 or #14 or #15 or #16 or #17 or #18 or #19 or #20 or #21 |
| 23 | (pocus):ti,ab,kw |
| 24 | (point-of-care):ti,ab,kw |
| 25 | ("point of care"):ti,ab,kw |
| 26 | (focus):ti,ab,kw (Word variations have been searched) |
| 27 | (fcu):ti,ab,kw |
| 28 | MeSH descriptor: [Point-of-Care Testing] this term only |
| 29 | MeSH descriptor: [Point-of-Care Systems] this term only |
| 30 | (protocol):ti,ab,kw |
| 31 | #23 or #24 or #25 or #26 or #27 or #28 or #29 or #30 |
| 32 | (ultraso):ti,ab,kw (Word variations have been searched) |
| 33 | (Echocardiogra):ti,ab,kw (Word variations have been searched) |
| 34 | MeSH descriptor: [Ultrasonography] this term only |
| 35 | MeSH descriptor: [Ultrasonics] this term only |
| 36 | #32 or #33 or #34 or #35 |
| 37 | MeSH descriptor: [Shock] this term only |
| 38 | MeSH descriptor: [Hypotension] this term only |
| 39 | (shock):ti,ab,kw |
| 40 | (hypotension):ti,ab,kw |
| 41 | ("circulatory failure"):ti,ab,kw |
| 42 | ("circulatory dysfunction"):ti,ab,kw |
| 43 | ("circulatory disorder"):ti,ab,kw (Word variations have been searched) |
| 44 | #37 or #38 or #39 or #40 or #41 or #42 or #43 |
| 45 | (#9 or #22) and #31 and #36 and #44 |
|  |  |
| Embase | |
| 1 | EMB.EXACT.EXPLODE("intensive care") |
| 2 | EMB.EXACT.EXPLODE("intensive care unit") |
| 3 | EMB.EXACT.EXPLODE("medical intensive care unit") |
| 4 | EMB.EXACT.EXPLODE("critical illness") |
| 5 | EMB.EXACT.EXPLODE("critical care outcome") |
| 6 | ti("intensive care") OR ab("intensive care") |
| 7 | ti("critical care") OR ab("critical care") |
| 8 | ti(critical AND ill*) OR ab(critical AND ill*) |
| 9 | ti(acute) OR ab(acute) |
| 10 | ti(emergen*) OR ab(emergen*) |
| 11 | ti(rapid) OR ab(rapid) |
| 12 | EMB.EXACT.EXPLODE("acute disease") |
| 13 | EMB.EXACT.EXPLODE("emergency health service") |
| 14 | EMB.EXACT.EXPLODE("emergency care") |
| 15 | EMB.EXACT.EXPLODE("emergency nursing") |
| 16 | EMB.EXACT.EXPLODE("emergency medicine") |
| 17 | EMB.EXACT.EXPLODE("emergency") |
| 18 | EMB.EXACT.EXPLODE("evidence based emergency medicine") |
| 19 | EMB.EXACT.EXPLODE("emergency treatment") |
| 20 | EMB.EXACT.EXPLODE("rapid response team") |
| 21 | S1 OR S2 OR S3 OR S4 OR S5 OR S6 OR S7 OR S8 OR S9 OR S10 OR S11 OR S12 OR S13 OR S14 OR S15 OR S16 OR S17 OR S18 OR S19 OR S20 |
| 22 | ti(POCUS) OR ab(POCUS) |
| 23 | ti(point-of-care) OR ab(point-of-care) |
| 24 | ti("point of care") OR ab("point of care") |
| 25 | ti(focus*) OR ab(focus*) |
| 26 | ti(FCU) OR ab(FCU) |
| 27 | ti(Protocol) OR ab(Protocol) |
| 28 | EMB.EXACT.EXPLODE("point of care testing") |
| 29 | EMB.EXACT.EXPLODE("point of care system") |
| 30 | S22 OR S23 OR S24 OR S25 OR S26 OR S27 OR S28 OR S28 OR S29 |
| 31 | ti(ultraso*) OR ab(ultraso*) |
| 32 | ti(ecocardiogra*) OR ab(ecocardiogra*) |
| 33 | EMB.EXACT.EXPLODE("ultrasound") |
| 34 | EMB.EXACT.EXPLODE("cardiovascular ultrasound system") |
| 35 | S31 OR S32 OR S33 OR S34 |
| 36 | S30 AND S35 |
| 37 | EMB.EXACT.EXPLODE("point of care ultrasound") |
| 38 | S36 OR S37 |
| 39 | ti(shock) OR ab(shock) |
| 40 | ti(hypotension) OR ab(hypotension) |
| 41 | ti("circulatory failure") OR ab("circulatory failure") |
| 42 | ti("circulatory dysfunction") OR ab("circulatory dysfunction") |
| 43 | ti("circulatory disorder*") OR ab("circulatory disorder*") |
| 44 | EMB.EXACT.EXPLODE("shock") |
| 45 | EMB.EXACT.EXPLODE("hypotension") |
| 46 | S39 OR S40 OR S41 OR S42 OR S43 OR S44 OR S45 |
| 47 | S21 AND S38 AND S46 |
|  |  |
| Web of Science | |
| 1 | (AB=("intensive care")) OR TI=("intensive care") |
| 2 | (TI=("critical ill*")) OR AB=("critical ill*") |
| 3 | (TI=("critical care")) OR AB=("critical care") |
| 4 | #1 OR #2 OR #3 |
| 5 | (TI=(acute)) AND AB=(acute) |
| 6 | (TI=(Emergen*)) OR AB=(Emergen*) |
| 7 | (TI=(rapid)) OR AB=(rapid) |
| 8 | #5 OR #6 OR #7 |
| 9 | #4 OR #8 |
| 10 | (TI=(pocus)) OR AB=(pocus) |
| 11 | (TI=(point-of-care)) OR AB=(point-of-care) |
| 12 | (TI=("point of care")) OR AB=("point of care") |
| 13 | (TI=(focus*)) OR AB=(focus*) |
| 14 | (TI=(fcu)) OR AB=(fcu) |
| 15 | #10 OR #11 OR #12 OR #13 OR #14 |
| 16 | (TI=(Ultraso*)) OR AB=(Ultraso*) |
| 17 | (TI=(Echocardiogra*)) OR AB=(Echocardiogra*) |
| 18 | #17 OR #16 |
| 19 | (TI=(shock)) OR AB=(shock) |
| 20 | (TI=(hypotension)) OR AB=(hypotension) |
| 21 | (TI=("circulatory failure")) OR AB=("circulatory failure") |
| 22 | (TI=("circulatory dysfunction")) OR AB=("circulatory dysfunction") |
| 23 | (TI=("circulatory disorder*")) OR AB=("circulatory disorder*") |
| 24 | #22 OR #23 OR #21 OR #20 OR #19 |
| 25 | #9 AND #15 AND #18 AND #24 |
|  |  |

**Table S2:** **The list of excluded studies**

| Study | Reason for exclusion |
| --- | --- |
| Rozycki 1998 [1] | Wrong study design |
| Moore 2002 [2] | Wrong study design |
| Atkinson 2019 [3] | Wrong study design |
| Zhou 2019 [4] | Wrong study design |
| Lafon 2020 [5] | Wrong study design |
| Lenz 2021 [6] | Wrong study design |
| Ienghong 2022 [7] | Wrong study design |
| Jones 2004 [8] | Wrong study design |
| Stachura 2014 [9] | Wrong study design |
| Dinc 2015 [10] | Wrong study design |
| Charron 2015 [11] | Wrong study design |
| Mesterházi 2016 [12] | Wrong study design |
| Daley 2016 [13] | Wrong study design |
| Sasmaz 2017 [14] | Wrong study design |
| Peach 2017 [15] | Wrong study design |
| Mitra 2013 [16] | Incomplete data: unknown contact information, conference abstract |
| Volpicelli 2013 [17] | Incomplete data: unanswered request for further information |
| Gunaydin 2016 [18] | Incomplete data: unanswered request for further information |
| Becker 2017 [19] | Incomplete data: unanswered request for further information |
| Mahmoud 2019 [20] | Incomplete data: unknown contact information, conference abstract |
| Leroux 2021 [21] | Incomplete data: unanswered request for further information |
| Ghane 2015 [22] | Duplicate: report in the mid-stage of a study that included (reference number 33 in the main text) |
| Daley 2019 [23] | Duplicate: same as Charron 2015 [11] in this table |
| Keefer 2021 [24] | Duplicate: same as reference number 32 in the main text |

**References**

1. Rozycki GS, Ballard RB, Feliciano DV, Schmidt JA, Pennington SD. Surgeon-performed ultrasound for the assessment of truncal injuries: Lessons learned from 1540 patients. Ann Surg. 1998;228:557–67.
2. Moore CL, Rose GA, Tayal VS, Sullivan DM, Arrowood JA, Kline JA. Determination of left ventricular function by emergency physician echocardiography of hypotensive patients. Acad Emerg Med. 2002;9:186–93.
3. Atkinson P, Peach M, Hunter S, Kanji A, Taylor L, Lewis D, et al. Does point-of-care ultrasonography improve diagnostic accuracy in emergency department patients with undifferentiated hypotension? An international randomized controlled trial from the SHoC-ED investigators. Can J Emerg Med. 2019;21:S15.
4. Zhou R, Yin W-H, Liu B-Y, Zou T-J, Li Y, Deng L-J, et al. The Clinical Value of the Pathophysiology Oriented Critical Care Ultrasound Exam (POCCUE) Protocol in Acute Respiratory and Circulatory Compromise of Critically Ill Patients. Sichuan Da Xue Xue Bao Yi Xue Ban. 2019;50:792–7.
5. Lafon T, Appert A, Hadj M, Bigrat V, Legarcon V, Claveries P, et al. Comparative early hemodynamic profiles in patients presenting to the emergency department with septic and nonseptic acute circulatory failure using focused echocardiography. Shock. 2020;53:695–700.
6. Lenz TJ, Phelan MB, Grawey T. Determining a Need for Point-of-Care Ultrasound in Helicopter Emergency Medical Services Transport. Air Med J. 2021;40(3):175-178.
7. Ienghong K, Cheung LW, Tiamkao S, Bhudhisawasdi V, Apiratwarakul K. The Utilization of Handheld Ultrasound Devices in a Prehospital Setting. Prehosp Disaster Med. 2022;37(4):355-359.
8. Jones AE, Tayal VS, Sullivan DM, Kline JA. Randomized, controlled trial of immediate versus delayed goal-directed ultrasound to identify the cause of nontraumatic hypotension in emergency department patients. Crit Care Med. 2004;32(8):1703-1708.
9. Stachura MR, Landes M, Venugopal R, Aklilu F, Sarrazin J, Azazh A. Evaluation of a point-of-care ultrasound scan list in the emergency department at Black Lion Hospital, Addis Ababa, Ethiopia. Can J Emerg Med. 2014;16(S1):S109.
10. Dinc SE, Soyuncu S, Dinc B, Oskay A, Bektas F. The effect of the emergency physicians’ clinical decision of targeted ultrasonography application in non-traumatic shock patients. Hong Kong J Emerg Med. 2015;22(6):364-370.
11. Charron C, Templier F, Goddet NS, Baer M, Vieillard-Baron A. Difficulties encountered by physicians in interpreting focused echocardiography using a pocket ultrasound machine in prehospital emergencies. Eur J Emerg Med. 2015;22:17-22.Mesterházi A, Barta M, Zubek L. Evaluation of the diagnostic value of bedside ultrasonography in the critical care. Orv Hetil. 2016;157:569-74. doi: 10.1556/650.2016.30320
12. Mesterházi A, Barta M, Zubek L. Evaluation of the diagnostic value of bedside ultrasonography in the critical care. Orv Hetil. 2016;157(14):569-574.
13. Daley J, Grotberg J, Pare J, Medoro A, Liu R, Hall MK, et al. Emergency physician performed tricuspid annular plane systolic excursion in the evaluation of suspected pulmonary embolism. Am J Emerg Med. 2017;35:106-11.
14. Sasmaz MI, Gungor F, Guven R, Akyol KC, Kozaci N, Kesapli M. Effect of Focused Bedside Ultrasonography in Hypotensive Patients on the Clinical Decision of Emergency Physicians. Emerg Med Int. 2017;2017:7020790.
15. Peach M, Milne J, Lewis D, Diegelmann L, Lamprecht H, Stander M, et al. Does point of care ultrasonography improve diagnostic accuracy in emergency department patients with undifferentiated hypotension? the first Sonography in Hypotension and Cardiac Arrest in the Emergency Department (SHOC-ED1) Study; An international random. Can J Emerg Med. 2018;20:157-64.
16. Mitra S, Appachi MS, Srinath TS, Chandrashekaran VP. Assessment of hypotension in non-traumatic patients in the emergency department: clinical versus RUSH protocol. Indian J Crit Care Med. 2013;17:17.
17. Volpicelli G, Lamorte A, Tullio M, Cardinale L, Giraudo M, Stefanone V, et al. Point-of-care multiorgan ultrasonography for the evaluation of undifferentiated hypotension in the emergency department. Intensive Care Med. 2013;39:1290-8.
18. Gunaydin I, Kekec Z, Ay MO. Effectiveness of ultrasound in hypotensive patients. Crit Ultrasound J. 2016;8:8.
19. Becker TK, Tafoya CA, Osei-Ampofo M, Tafoya MJ, Kessler RA, Theyyunni N, et al. Cardiopulmonary ultrasound for critically ill adults improves diagnostic accuracy in a resource-limited setting: the AFRICA trial. Trop Med Int Health. 2017;22:1599-608.
20. Mahmoud HA, Agamy GMR, Ahmad AM, Ahmad EF. Role of RUSH protocol in management of shocked patients. Eur Respir J. 2019;54:PA4235.
21. Leroux P, Javaudin F, Le Bastard Q, Lebret Y, Pes P, Arnaudet I, et al. Goal-directed ultrasound protocol in patients with nontraumatic undifferentiated shock in the emergency department: prospective dual centre study. Eur J Emerg Med. 2021;28:306-11.
22. Ghane MR, Gharib M, Ebrahimi A, Saeedi M, Akbari-Kamrani M, Rezaee M, et al. Accuracy of early rapid ultrasound in shock (RUSH) examination performed by emergency physician for diagnosis of shock etiology in critically ill patients. J Emerg Trauma Shock. 2015;8:5-10.
23. Daley JI, Dwyer KH, Grunwald Z, Shaw DL, Stone MB, Schick A, et al. Increased Sensitivity of Focused Cardiac Ultrasound for Pulmonary Embolism in Emergency Department Patients With Abnormal Vital Signs. Acad Emerg Med. 2019;26:1211–20.
24. Keefer S, Atkinson P, Chandra K, Henneberry RJ, Olszynski PA, Peach M, et al. Sonographic Findings of Left Ventricular Dysfunction to Predict Shock Type in Undifferentiated Hypotensive Patients: An Analysis From the Sonography in Hypotension and Cardiac Arrest in the Emergency Department (SHoC-ED) Study. Cureus. 2021;13:e16360.

**Table S3:** **Additional study characteristics**

| Study, Year | Conflict of interest | Funding source | Suspected disease | Timing of US diagnosis | Training program for physician | Training program |
| --- | --- | --- | --- | --- | --- | --- |
| Bagheri-Hariri et al, 2015 | None | None | None | During the initial management in the ED | Unclear |  |
| Ghane et al, 2015 | Unclear | A grant from the trauma research center of Baqiyatallah University of Medical Sciences. | None | At the time of the patients’ arrival | Yes | A 20-hour workshop for emergency ultrasound including the RUSH exam |
| Shokoohi et al, 2015 | None | Institutional departmental funds at the Department of Emergency Medicine, George Washington University, Washington, DC. | None | In the ED after enrolling patients | Unclear |  |
| Agmy et al, 2017 | None | Unclear | None | During the initial management for shock patients | Unclear |  |
| Nazerian et al, 2017 | None | Unclear | Yes (PE) | Immediately upon the possibility of a PE diagnosis within 3 h from inclusion | No |  |
| Elbaih et al, 2018 | Unclear | Unclear | None | During the initial management in the ED | Unclear |  |
| Tesfaye et al, 2018 | Unclear | Unclear | None | During the initial management in the ED | Unclear |  |
| Daley et al, 2019 | None | None | Yes (PE) | During the initial management in the ED | Yes | All personnel received standardized training that consisted of a brief video and a 1-hour didactic meeting to ensure that standardized images were being obtained. Two of the resident physicians underwent an additional didactic session conducted by an ultrasound fellowship–trained emergency physician. Residents later performed supervised practice examinations until the ultrasound fellowship–trained emergency physician was satisfied that they could reliably perform all the components of FOCUS prior to enrolling patients in the study. These residents were PGY-3 in emergency medicine and had prior ultrasound experience consistent with their level of residency training. The third resident, the primary author, already had significant experience in FOCUS (including TAPSE) and did not undergo additional training for study purposes. The three medical students underwent a 1-hour didactic and 1-hour hands-on training session by the primary author. Medical students were in their third year of medical school and did not have significant experience in bedside ultrasound prior to becoming involved in this study. Each student then completed 20 FOCUS examinations with feedback under the supervision of the primary author prior to enrolling patients in the study |
| Rahulkumar et al, 2019 | None | None | None | During the initial management in the ED | Unclear |  |
| Javali et al, 2020 | None | None | None | During the initial management in the ED | Unclear |  |
| Keefer et al, 2021 | None | None | None | Within the ﬁrst 60 minutes of the patient visit | Yes | Unclear |
| Zieleskiewicz et al, 2021 | Two authors received fees from General Electric Healthcare for ultrasound teaching | None | None | During the initial management as a rapid response team | Yes | The three-hour training session for typical ultrasound signs of the main causes of acute respiratory and circulatory failure, followed by classroom presentation of practical interactive case reports and a hands-on scenario-based workshop. |

PE, pulmonary embolism; US, ultrasound; ED, emergency department; RUSH, rapid ultrasound for shock and hypotension; FOCUS, focused cardiac ultrasound; PGY, post graduate year TAPSE, tricuspid annular plane systolic excursion.

**Table S4:** **Diagnostic accuracies for studies conducted in emergency department**

| Shock type | No. of Patients (study) | Sensitivity | Specificity | Area under the ROC curve | Positive  likelihood ratio | Negative likelihood ratio |
| --- | --- | --- | --- | --- | --- | --- |
| Obstructive | 725 (7) | 0.83 (0.65 - 0.92) | 0.97 (0.87 - 0.99) | 0.95 (0.77 - 0.98) | 41 (8.7 - 124) | 0.19 (0.08 - 0.36) |
| Cardiogenic | 743 (7) | 0.75 (0.49 - 0.90) | 0.94 (0.88 - 0.97) | 0.95 (0.83 - 0.97) | 16 (5.1 - 35) | 0.29 (0.10 - 0.57) |
| Hypovolemic | 602 (7) | 0.91 (0.84 - 0.95) | 0.92 (0.85 - 0.96) | 0.96 (0.89 - 0.96) | 13 (7.2 - 21) | 0.10 (0.05 - 0.17) |
| Distributive | 509 (6) | 0.78 (0.69 - 0.84) | 0.98 (0.95 - 0.99) | 0.95 (0.79 - 0.98) | 31 (15 - 57) | 0.23 (0.16 - 0.32) |

**Table S5:** **Diagnostic accuracies for studies with no prior suspected disease**

| Shock type | No. of Patients (study) | Sensitivity | Specificity | Area under the ROC curve | Positive  likelihood ratio | Negative likelihood ratio |
| --- | --- | --- | --- | --- | --- | --- |
| Obstructive | 569 (7) | 0.87 (0.77 - 0.93) | 0.98 (0.96 - 0.99) | 0.98 (0.82 - 0.98) | 50 (23 - 196) | 0.14 (0.07 - 0.24) |

**Table S6:** **Diagnostic accuracies for studies in which the existence of a point of care ultrasound training program was explicitly described**

| Shock type | No. of Patients (study) | Sensitivity | Specificity | Area under the ROC curve | Positive  likelihood ratio | Negative likelihood ratio |
| --- | --- | --- | --- | --- | --- | --- |
| Obstructive | 227 (3) | 0.88 (0.75 - 0.95) | 0.92 (0.54 - 0.99) | 0.90 (0.79 - 0.97) | 19 (2.0 - 90) | 0.14 (0.06 - 0.29) |
| Cardiogenic | 226 (3) | 0.77 (0.49 - 0.92) | 0.96 (0.90 - 0.98) | 0.96 (0.70 - 0.98) | 19 (6.0 - 44) | 0.26 (0.09 - 0.55) |
| Hypovolemic | 91 (2) | 0.95 (0.70 - 0.99) | 0.96 (0.88 - 0.99) | 0.98 (0.90 - 0.99) | 26 (7.3 - 68) | 0.08 (0.01 - 0.32) |
| Distributive | 91 (2) | 0.82 (0.51 - 0.95) | 0.98 (0.77 - 1.0) | 0.96 (0.76 - 0.98) | 72 (3.7 - 375) | 0.21 (0.05 - 0.50) |

**Table S7:** **Diagnostic accuracies for studies in which ultrasound other than transthoracic echocardiography was used in combination**

| Shock type | No. of Patients (study) | Sensitivity | Specificity | Area under the ROC curve | Positive  likelihood ratio | Negative likelihood ratio |
| --- | --- | --- | --- | --- | --- | --- |
| Obstructive | 674 (8) | 0.82 (0.65 - 0.92) | 0.98 (0.96 - 0.99) | 0.99 (0.83 - 0.98) | 52 (24 - 100) | 0.19 (0.09 - 0.36) |

**Table S8:** **Diagnostic accuracies for studies without high risk of bias studies**

| Shock type | No. of Patients (study) | Sensitivity | Specificity | Area under the ROC curve | Positive  likelihood ratio | Negative likelihood ratio |
| --- | --- | --- | --- | --- | --- | --- |
| Obstructive | 747 (8) | 0.82 (0.66 - 0.92) | 0.97 (0.89 - 0.99) | 0.95 (0.77 - 0.97) | 80 (38 - 107) | 0.19 (0.09 - 0.35) |
| Cardiogenic | 765 (8) | 0.75 (0.51 - 0.89) | 0.95 (0.89 - 0.97) | 0.95 (0.84 - 0.97) | 16 (5.8 - 34) | 0.28 (0.11 - 0.53) |
| Hypovolemic | 531 (7) | 0.89 (0.82 - 0.95) | 0.93 (0.86 - 0.97) | 0.96 (0.86 - 0.96) | 14 (7.5 - 25) | 0.11 (0.06 - 0.19) |
| Distributive | 531 (7) | 0.79 (0.71 - 0.85) | 0.97 (0.95 - 0.99) | 0.96 (0.77 - 0.97) | 30 (15 - 54) | 0.22 (0.16 - 0.30) |

**Figure S1: Obstructive shock**


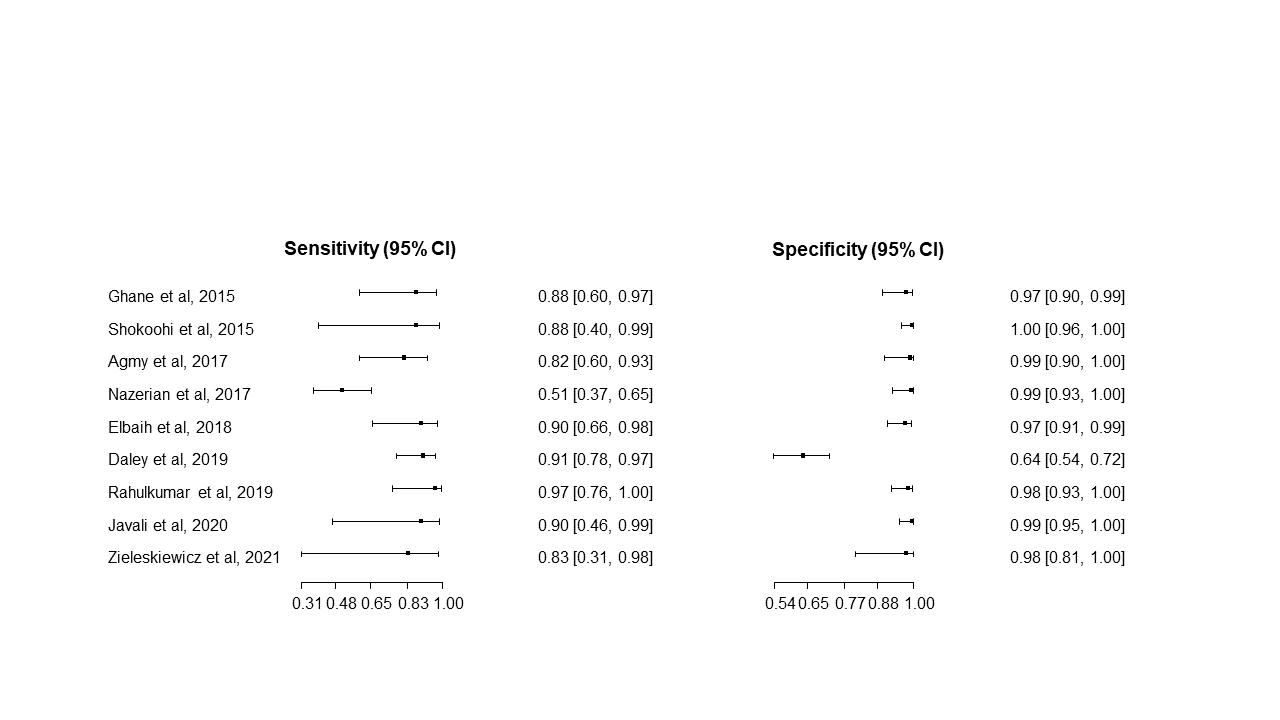


**Figure S2: Cardiogenic shock**


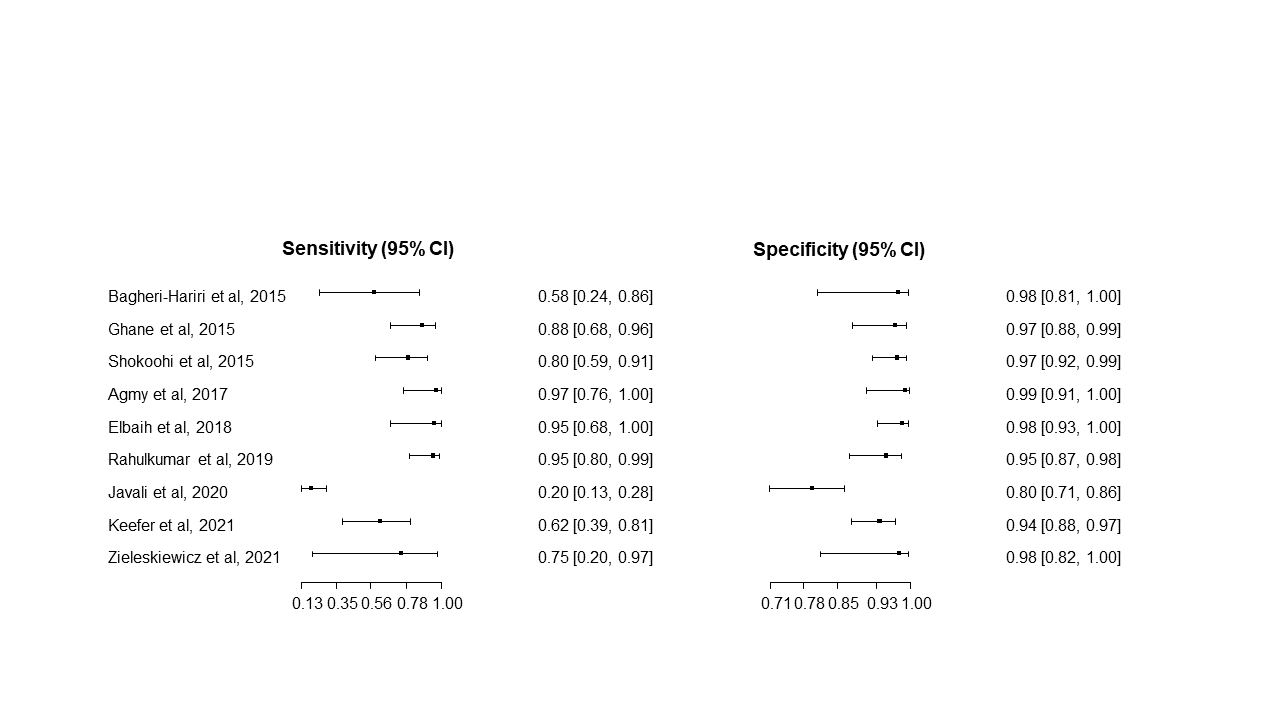


**Figure S3: Hypovolemic shock**


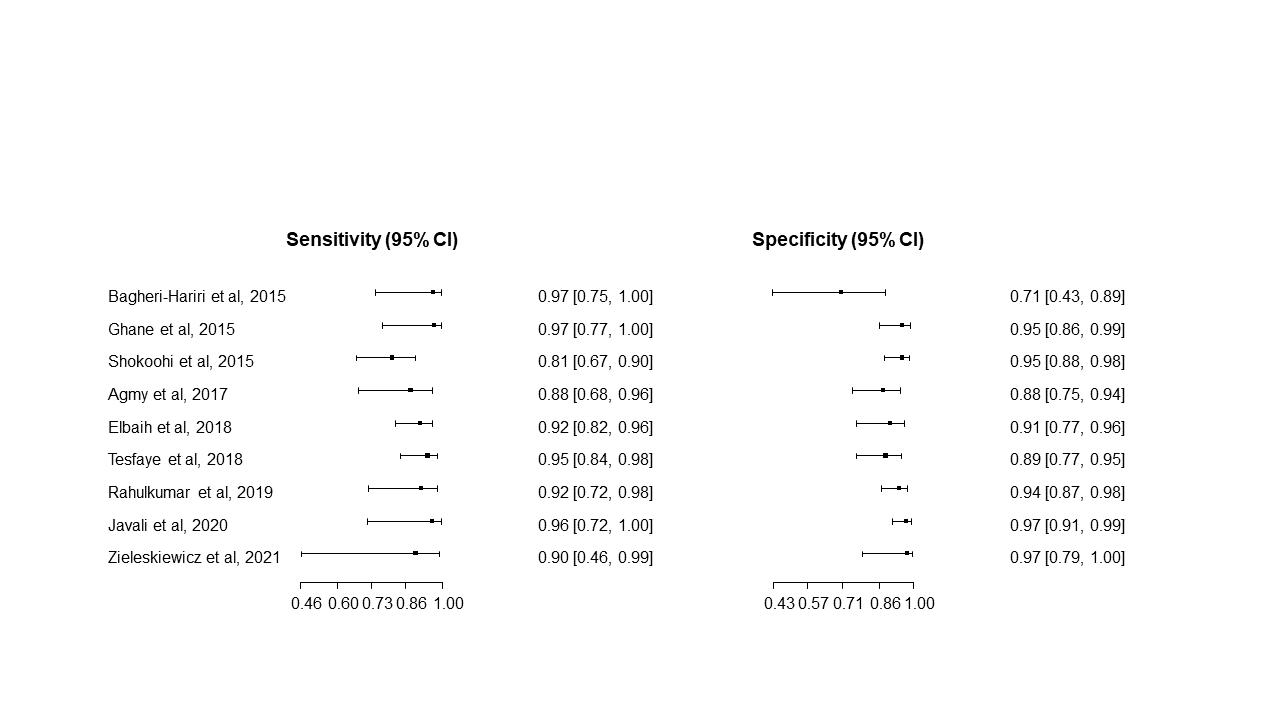


**Figure S4: Distributive shock**


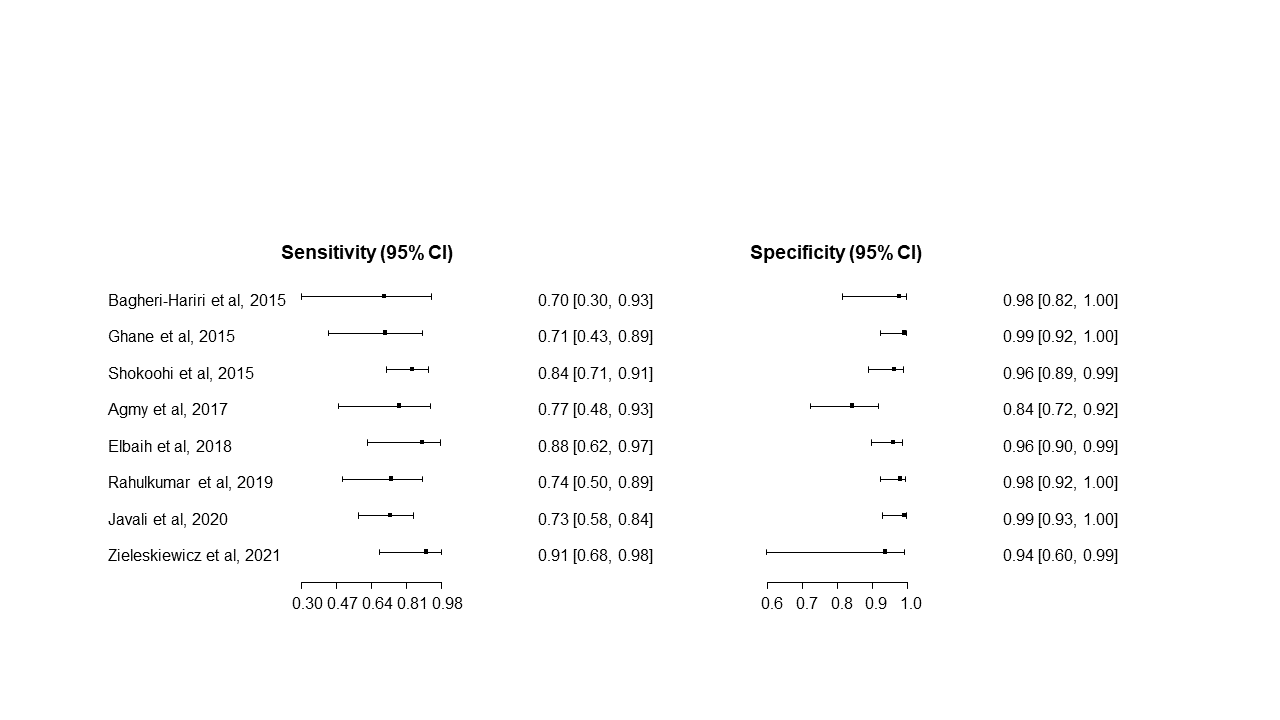


**Figure S5: Mixed shock**


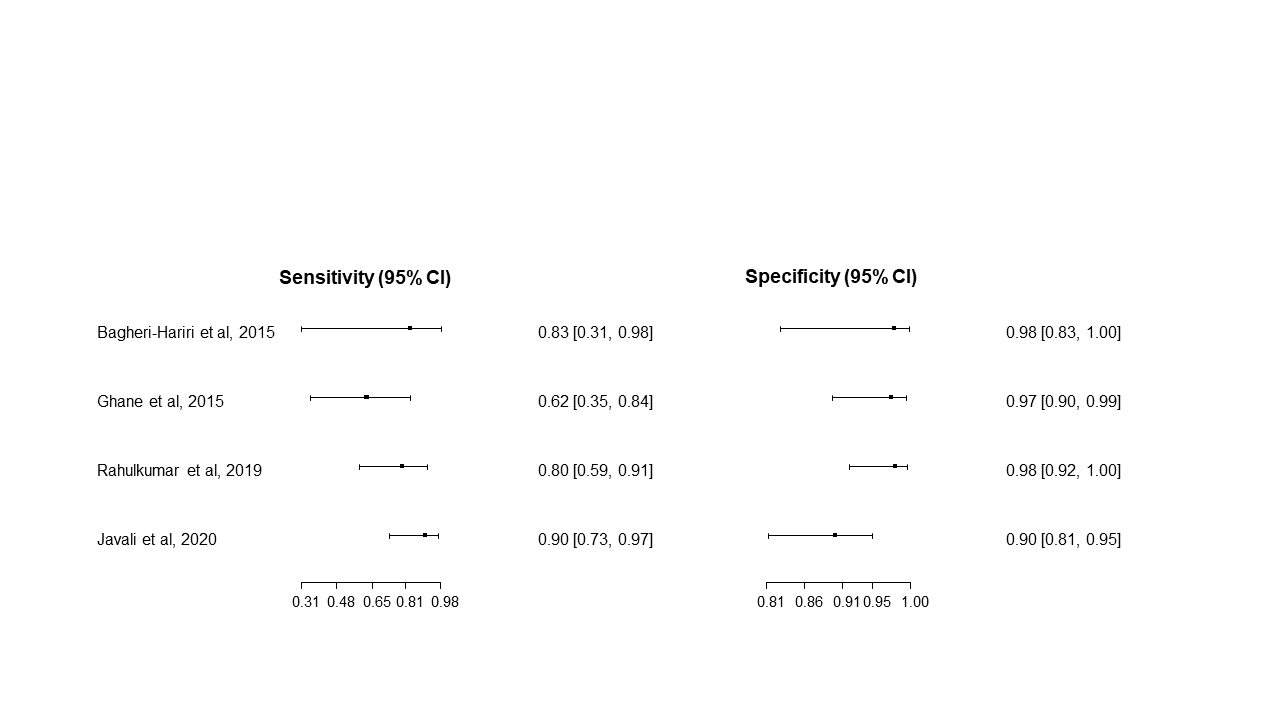

Supplement: Supplementary file 1 — Additional file 1: Table S1. The full search strategy. Table S2. The list of excluded studies. Table S3. Additional study characteristics. Table S4. Diagnostic accuracies for studies conducted in the emergency department. Table S5. Diagnostic accuracies for studies with no prior suspected disease. Table S6. Diagnostic accuracies for studies in which the existence of a point-of-care ultrasound training program was explicitly mentioned. Table S7. Diagnostic accuracies for studies in which ultrasound other than transthoracic echocardiography was used in combination. Table S8. Diagnostic accuracies for studies without high risk of bias. Figure S1: Obstructive shock. CI, confidence interval. Figure S2: Cardiogenic shock. CI, confidence interval. Figure S3: Hypovolemic shock. CI, confidence interval. Figure S4: Distributive shock. CI, confidence interval. Figure S5: Mixed shock. CI, confidence interval. [file 13054_2023_4495_MOESM1_ESM.docx]
